# Accelerated epigenetic aging and decreased natural killer cells based on DNA methylation in patients with untreated major depressive disorder

**DOI:** 10.1038/s41514-023-00117-1

**Published:** 2023-09-06

**Authors:** Ryota Shindo, Takaki Tanifuji, Satoshi Okazaki, Ikuo Otsuka, Toshiyuki Shirai, Kentaro Mouri, Tadasu Horai, Akitoyo Hishimoto

**Affiliations:** Department of Psychiatry, Kobe University Graduate School of Medicine, Kobe, Japan

**Keywords:** Epigenetics, Psychiatric disorders

## Abstract

Major depressive disorder (MDD) is known to cause significant disability. Genome-wide DNA methylation (DNAm) profiles can be used to estimate biological aging and as epigenetic clocks. However, information on epigenetic clocks reported in MDD patients is inconsistent. Since antidepressants are likely confounders, we evaluated biological aging using various DNAm-based predictors in patients with MDD who had never received depression medication. A publicly available dataset consisting of whole blood samples from untreated MDD patients (*n* = 40) and controls (*n* = 40) was used. We analyzed five epigenetic clocks (HorvathAge, HannumAge, SkinBloodAge, PhenoAge, and GrimAge), DNAm-based telomere length (DNAmTL), and DNAm-based age-related plasma proteins (GrimAge components), as well as DNAm-based white blood cell composition. The results indicate that patients with untreated MDD were significantly associated with epigenetic aging acceleration in HannumAge and GrimAge. Furthermore, a decrease in natural killer cells, based on DNAm, was observed in patients with untreated MDD.

## Introduction

Major depressive disorder (MDD) is a prevalent mental illness and is considered the leading cause of disability among psychiatric diseases worldwide, affecting an estimated 280 million individuals^1^. Depression has been linked to an increased risk of premature mortality from various comorbidities and all-cause mortality, including suicide^2^. Given that telomere shortening is a marker of cellular aging, depression has been found to be linked to accelerated biological aging in terms of telomere length^3,4^ and leads to neuropsychiatric disease-related aging such as dementia^5,6^. However, previous studies have shown that antidepressant medication prevents telomere shortening and the development of dementia^6–8^.

Recently, some researchers developed epigenetic clocks using genome-wide DNA methylation (DNAm) profiles to estimate biological aging^9–13^. Moreover, DNAm-based telomeres length (DNAmTL) and DNAm-based white blood cell compositions, which are associated with biological aging, have also been created^10,14,15^. The five epigenetic clocks, HorvathAge, HannumAge, SkinBloodAge, PhenoAge, and GrimAge as well as DNAmTL have been widely used^9–14^. Epigenetic clocks have been evaluated in various conditions, including psychiatric disorders, as follows: patients with coronavirus disease 2019^16^, cancers^17^, cardiovascular disease^18^, diabetes^19^, MDD^20–25^, bipolar disorder^26^, schizophrenia^27^, post-traumatic stress disorder^28^, suicide^29^, and all-cause mortality^30^. The five epigenetic clocks have been utilized in the studies of various psychiatric disorders to provide an accurate estimation through various unique CpG sites^31^.

Epigenetic clocks have been previously evaluated in patients with MDD, but the results remain inconsistent^20–23,25,32,33^. Protsenko et al. reported that patients with untreated MDD exhibited greater epigenetic aging acceleration than healthy controls, using GrimAge^23^. Additionally, a previous study demonstrated that maternal prenatal antidepressant use can significantly decelerate offspring epigenetic age^34^. We hypothesized that depression is associated with accelerated biological aging based on DNAm, but that the use of depression medication may modulate changes in the methylation of CpG sites in patients with MDD. This could potentially confound the relationships between depression and biological aging markers. To verify our hypothesis, we analyzed five epigenetic clocks (HorvathAge, HannumAge, SkinBloodAge, PhenoAge, and GrimAge) and DNAmTL in patients with MDD who had never been treated with depression medication, as well as in control participants. We also evaluated the GrimAge components indicating age-predictive factors based on DNAm and DNAm-based white blood cell composition.

## Results

### Five epigenetic clocks, DNAmTL, and GrimAge components

**Fig. 1:**
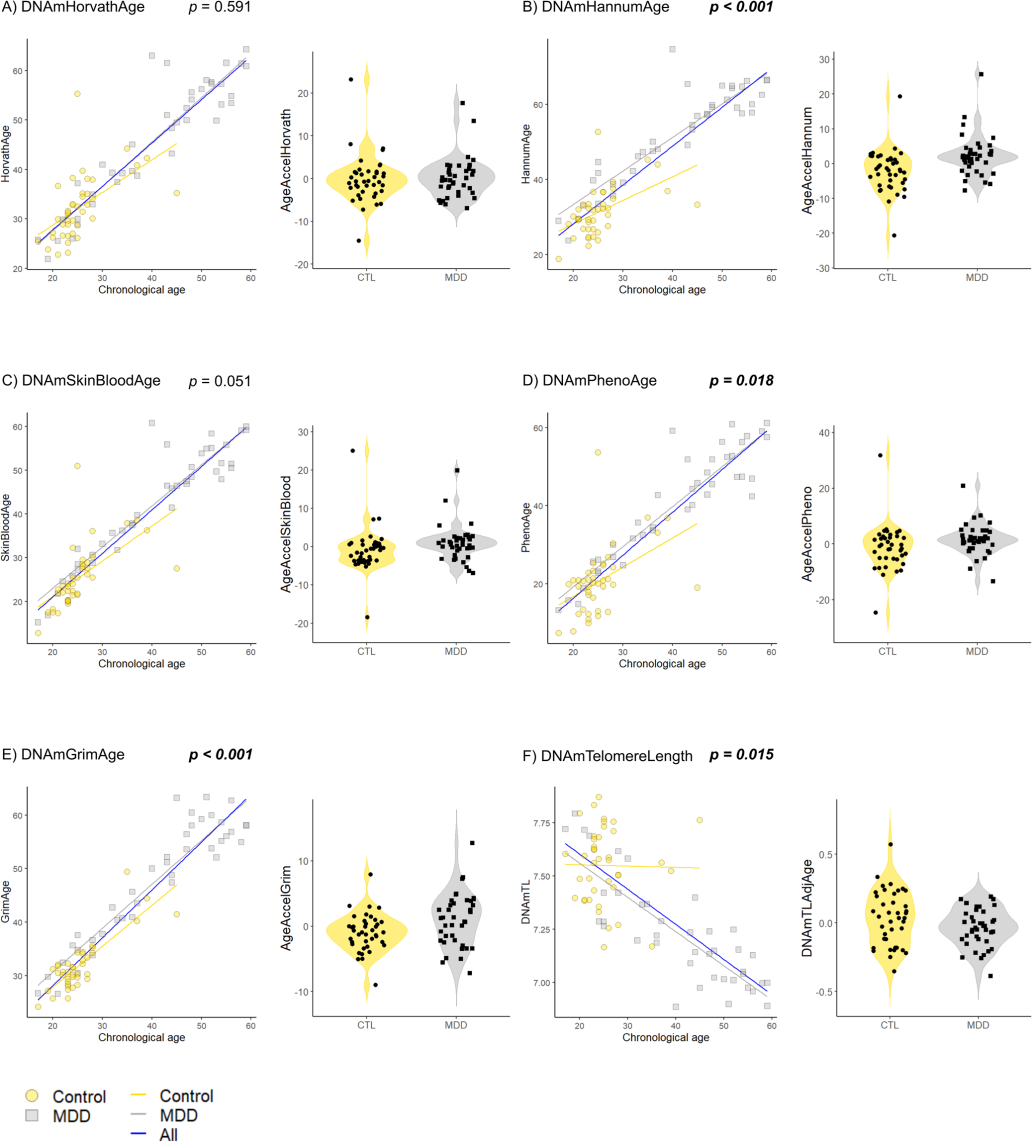
Comparison of epigenetic age and DNAmTL between patients with untreated MDD and controls. (**A**) HorvathAge, (**B**) HannumAge, (**C**) SkinBloodAge, (**D**) PhenoAge, (**E**) GrimAge, and (**F**) DNAmTL. The scatter plots indicate the epigenetic age and DNAmTL on the *y*-axis vs. the chronological age on the *x*-axis. We demonstrate the acceleration of epigenetic age and DNAmTLadjAge in patients with untreated MDD and in the control groups using the violin plots with data points. We refer *p*-values to multiple linear regression analysis. If *p*-values are significant at <0.05, they are denoted in bold and italics. CTL, control; DNAm, DNA methylation; DNAmTL, DNAm-based telomere length; DNAmTLadjAge, age-adjusted estimate of DNAm-based TL; MDD, major depressive disorder.

Participants’ demographic data, including sex and age, are presented in Table 1. Supplementary Tables 1, 2, 3 summarize the correlations between chronological age and each epigenetic clock/GrimAge component/DNAm-based white blood cell composition (Fig. 1, and Supplementary Figs. 1, 2).

**Table 1:** Demographics, DNAm-based age acceleration and telomere length acceleration, as well as GrimAge components, and DNAm-based white blood cell counts between patients with untreated MDD and controls.

	CTL (*n* = 40)	MDD (*n* = 40)	*P*-value
*Demographic characteristics*			
Sex (male/female)	20/20	16/24	0.106^a^
Age (years old), median (IQR)	24.0 (23.0, 26.25)	43.5 (29.5, 52.0)	***<0.001***^***b***^
*DNAmAge and DNAmTL acceleration*^c^			
AgeAccelHorvath, median (IQR)	−0.69 (−2.94, 1.49)	−0.07 (−2.42, 2.15)	0.684^b^
AgeAccelHannum, median (IQR)	−1.86 (−5.75, 1.21)	1.75 (−0.002, 3.72)	***<0.001***^***b***^
AgeAccelSkinBlood, median (IQR)	−0.10 (−3.67, 0.59)	0.70 (−0.68, 2.19)	***0.0098***^***b***^
AgeAccelPheno, median (IQR)	−1.30 (−5.19, 2.31)	1.30 (−1.57, 3.36)	***0.0333***^***b***^
AgeAccelGrim, median (IQR)	−0.97 (−2.51, 0.43)	1.30 (−2.14, 3.61)	***0.013***^***b***^
DNAmTLadjAge, median (IQR)	0.04 (−0.14, 0.20)	−0.03 (−0.12, 0.07)	0.0914^b^
*GrimAge components*^d^			
DNAmADM [pg/ml] (IQR)	278.378 (262.456, 291.612)	317.582 (300.479, 337.206)	***<0.001***^***b***^
DNAmB2M [pg/ml] (IQR)	1115022 (1071350, 1169213)	1440094 (1225107, 1549209)	***<0.001***^***b***^
DNAmCystatinC [pg/ml] (IQR)	504475 (486991, 515874)	589892 (539036, 605227)	***<0.001***^b^
DNAmGDF-15 [pg/ml] (IQR)	275.698 (226.861, 337.268)	523.425 (378.453, 622.928)	***<0.001***^***b***^
DNAmLeptin [pg/ml] (IQR)	4987.87 (−1390.06, 6282.70)	5711.41 (554.882, 7582.69)	***0.012***^***b***^
DNAmPAI-1 [pg/ml] (IQR)	12689.9 (11927.0, 14153.7)	15438.6 (13629.0, 17392.3)	***<0.001***^b^
DNAmTIMP-1 [pg/ml] (IQR)	28674.7 (28312.8 29072.3)	31910.2 (29996.9, 33285.2)	***<0.001***^***b***^
DNAmPACKYRS (IQR)	5.08330 (2.68010, 6.73291)	10.9202 (8.12618, 14.8601)	***<0.001***^b^
*DNAm-based white blood cell composition*^e^			
CD8+ T cell (IQR)	0.085 (0.058, 0.112)	0.095 (0.082, 0.129)	***0.031***^***b***^
Naive CD8+ T cell (IQR)	292.1 (256.1, 321.7)	230.7 (194.5, 271.4)	***<0.001***^***b***^
Exhausted CD8+ T cell (IQR)	9.33 (7.184, 11.934)	9.846 (8.739, 11.546)	0.595^b^
CD4+ T cell (IQR)	0.133 (0.097, 0.157)	0.111(0.075, 0.142)	0.058^b^
Naive CD4+ T cell (IQR)	422.0 (331.4, 473.0)	371.2 (323.2, 466.4)	0.524^b^
Natural killer cell (IQR)	0.130 (0.089, 0.163)	0.030 (0.018, 0.049)	***<0.001***^***b***^
Monocyte (IQR)	0.056 (0.048, 0.072)	0.059 (0.050, 0.072)	0.402^b^
Granulocyte (IQR)	0.548 (0.490, 0.595)	0.625 (0.554, 0.680)	***<0.001***^***b***^
B cell (IQR)	0.045 (0.037, 0.058)	0.049 (0.033, 0.063)	0.642^b^
Plasmablast (IQR)	1.817 (1.691, 1.962)	1.831 (1.692, 1.936)	0.792^b^

^a^We assessed *p* -value using the χ^2^-test.

^b^We assessed *p* -value using the Mann–Whitney U test.

^c^We defined epigenetic age acceleration (AgeAccelHorvath, AgeAccelHannum, AgeAccelSkinBlood, AgeAccelPheno, and AgeAccelGrim) as the residual from regressing each DNAm age on the chronological age. We defined DNAmTLadjAge as the residual from regressing DNAmTL on chronological age.

^d^GrimAge components indicated DNAm-based age-predictive factors.

^e^These white blood cell counts are predicted by DNAm.

If *p*-values are significant at <0.05, they are denoted in bold and in italics.

*ADM* adrenomedullin, *B2M* beta-2-microglobulin, *CD4* cluster of differentiation 4, *CD8* cluster of differentiation 8, *CTL* control, *DNAm* DNA methylation, *DNAmPACKYRS* DNA methylation-based smoking pack-years, *DNAmTL* DNA methylation-based telomere length, *DNAmTLadjAge* age-adjusted estimate of DNA methylation-based telomere length, *GDF-15* growth differentiation factor 15, *IQR* interquartile range, *MDD* major depressive disorder, *PAI-1* plasminogen activator inhibitor-1, *TIMP-1* tissue inhibitor ofmetalloproteinases-1.

Patients with untreated MDD showed significant age acceleration compared with controls in AgeAccelHannum (*p* < 0.001), AgeAccelSkinBlood (*p* = 0.0098), AgeAccelPheno (*p* = 0.0333), and AgeAccelGrim (*p* = 0.013) (Fig. 1 and Table 1). After adjusting for confounding factors, such as age and sex, significant differences were observed in AgeAccelHannum (*p* < 0.001, *R*^2^ = 0.197), AgeAccelPheno (*p* = 0.018, *R*^2^ = 0.045), AgeAccelGrim (*p* < 0.001, *R*^2^ = 0.376), and DNAmTLadjAge (*p* = 0.015, *R*^2^ = 0.092) (Fig. 1 and Table 2).

**Table 2:** Multiple regression analyses of DNAm-based Ages and TL acceleration, as well as GrimAge components, and DNAm-based white blood cell counts between patients with untreated MDD and controls.

	Phenotype		Age		Sex		Adjusted *R*^2^
	Estimate	*P*-value	Estimate	*P*-value	Estimate	*P*-value	
*DNAmAge and DNAmTL acceleration*^a^							
AgeAccelHorvath	0.80055684	0.591	−0.01499872	0.803	−1.69811179	0.150	−0.00887
AgeAccelHannum	7.3704388	***<0.001***	−0.1807105	***0.007***	−1.9114904	0.135	0.19710
AgeAccelSkinBlood	2.97353317	0.051	−0.07232145	0.236	−0.95934000	0.418	0.01747
AgeAccelPheno	4.717704	***0.018***	−0.114084	0.153	−1.734133	0.263	0.04549
AgeAccelGrim	3.56851612	***<0.001***	−0.07863095	***0.020***	−3.77890619	***<0.001***	0.37590
DNAmTLAdjAge	−0.11769426	***0.015***	0.00270866	0.160	0.08750912	***0.021***	0.09222
*GrimAge components*^b^							
DNAmADM	14.007312	***0.00012***	1.527318	***<0.001***	20.452635	***<0.001***	0.8329
DNAmB2M	53507.20	0.085	14468.58	***<0.001***	18964.53	0.434	0.7744
DNAmCystatinC	14627.800	***0.00536***	3640.076	***<0.001***	−4098.721	0.310	0.8876
DNAmGDF-15	58.962437	0.150	9.885624	***<0.001***	−1.349210	0.966	0.4982
DNAmLeptin	760.31512	0.235	36.74497	0.156	7059.14737	***<0.001***	0.7330
DNAmPAI-1	2319.41380	***0.000587***	32.76313	0.213	−1446.18057	***0.0057***	0.3066
DNAmTIMP-1	452.4535	***0.000149***	149.7430	***<0.001***	−229.8033	***0.0185***	0.9627
DNAmPACKYRS	3.3794458	0.123	0.3583494	***<0.001***	−8.5257449	***<0.001***	0.4356
*DNAm-based white blood cell composition*^c^							
CD8+ T cell	0.04056	***0.0013***	−0.00116	0.020	0.01363	0.157	0.1220
Naive CD8+ T cell	−14.754	0.221	−2.3271	***<0.001***	4.4057	0.641	0.3856
Exhausted CD8+ T cell	0.03132	0.973	0.02144	0.572	−0.27588	0.709	−0.0304
CD4+ T cell	−0.02286	0.146	−0.00009	0.882	0.00582	0.635	0.0135
Naive CD4+ T cell	27.370	0.333	−3.1214	***<0.001***	33.357	0.136	0.0848
Natural killer cell	−0.08937	***<0.001***	−0.00003	0.961	−0.01051	0.383	0.4169
Monocyte	0.01141	0.023	−0.00044	0.030	−0.00633	0.106	0.0681
Granulocyte	0.04956	0.070	0.00155	0.159	−0.00060	0.977	0.1246
B cell	0.00140	0.828	0.00006	0.819	−0.00009	0.986	−0.0358
Plasmablast	0.00564	0.914	−0.00059	0.779	−0.04562	0.268	−0.0210

Dummy variables: phenotype, CTL = 0, and MDD = 1; sex, male = 0 and female= 1.

If *p*-values are significant at <0.05, they are denoted in bold and in italics.

*R*^2^ is the coefficient of determination.

^a^We defined epigenetic age acceleration (AgeAccelHorvath, AgeAccelHannum, AgeAccelSkinBlood, AgeAccelPheno, and AgeAccelGrim) as the residual from regressing each DNAm age on the chronological age. We defined DNAmTLadjAge as the residual from regressing DNAmTL on chronological age.

^b^GrimAge components indicated DNAm-based age-predictive factors. For the GrimAge components, we corrected the significance level for multiple comparisons and the Bonferroni method defined the *p*-value as 0.05/8 = 0.00625 (eight GrimAge components).

If *p*-values are significant at <0.00625, they are denoted in bold and in italics.

^c^These white blood cell counts were predicted using DNAm. For DNAm-based white blood cells, we adjusted the significance level for multiple comparisons and the Bonferroni method defined the *p*-value as 0.05/10 = 0.005 (ten white blood cell compositions).

If *p*-values are significant at <0.005, they are denoted in bold and in italics.

*ADM* adrenomedullin, *B2M* beta-2-microglobulin, *CD4* cluster of differentiation 4, *CD8* cluster of differentiation 8, *CTL* control, *DNAm* DNA methylation, *DNAmPACKYRS* DNA methylation-based smoking pack-years, *DNAmTL* DNA methylation-based telomere length, *DNAmTLadjAge* age-adjusted estimate of DNA methylation-based telomere length, *GDF-15* growth differentiation factor 15, *MDD* major depressive disorder, *PAI-1* plasminogen activator inhibitor-1, *TIMP-1* tissue inhibitor ofmetalloproteinases-1.

**Fig. 2:**
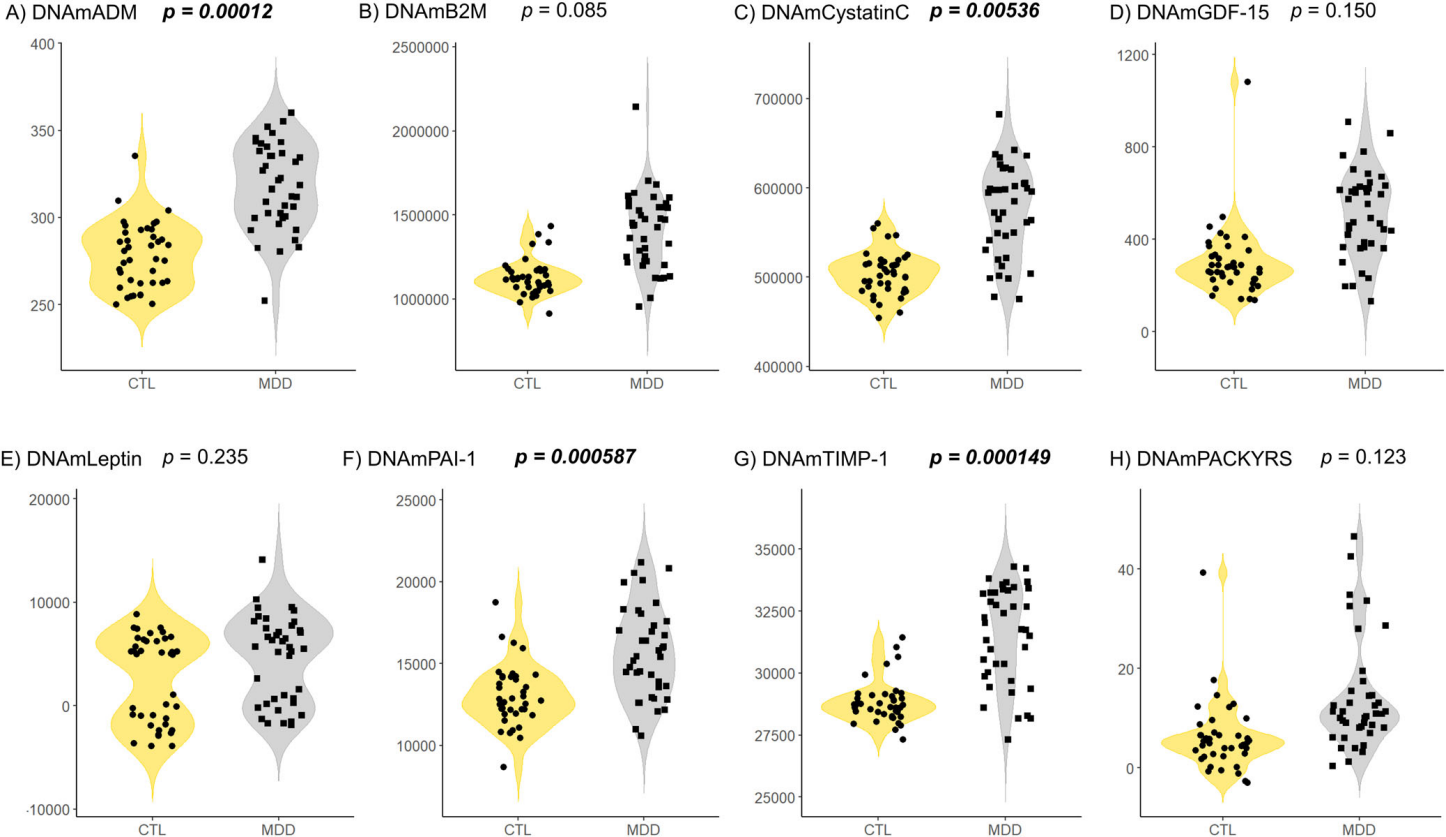
Comparison of GrimAge components between patients with untreated MDD and controls. (**A**) ADM, (**B**) B2M, (**C**) Cystatin C, (**D**) GDF-15, (**E**) Leptin, (**F**) PAI-1, (**G**) TIMP-1, and (**H**) PACKYRS. We show GrimAge components between patients with untreated MDD and control groups using the violin plots with data points. GrimAge components mean DNAm-based age-predictive factors. We refer *p*-values to multiple linear regression analysis. We corrected the significance level for multiple comparisons and the Bonferroni method defined the *p*-value as 0.05/8 = 0.00625. If *p*-values are significant at <0.00625, they are denoted in bold and italics. ADM, adrenomedullin; BMI, body mass index; B2M, beta-2-microglobulin; CTL, control; DNAm, DNA methylation; DNAmPACKYRS, DNA methylation-based smoking pack-years; GDF-15, growth differentiation factor 15; MDD, major depressive disorder; PACKYRS, smoking pack-years; PAI-1, plasminogen activator inhibitor-1; TIMP-1, tissue inhibitor ofmetalloproteinases-1.

All GrimAge components were significantly different between patients with untreated MDD and controls (Fig. 2 and Table 1). After adjusting for confounding factors such as age and sex, DNAmADM (*p* = 0.00012, *R*^2^ = 0.833), DNAmCystatinC (*p* = 0.00536, *R*^2^ = 0.888), DNAmPAI-1 (*p* = 0.000587, *R*^2^ = 0.307), and DNAmTIMP-1 (*p* = 0.000149, *R*^2^ = 0.963) remained significantly increased in patients with untreated MDD compared to those in controls (Fig. 2 and Table 2). These results withstood the Bonferroni correction for multiple comparisons of eight GrimAge components (corrected significance was defined as *p*-value < 0.05/8 = 0.00625).

### White blood cell composition predicted based on DNAm

**Fig. 3:**
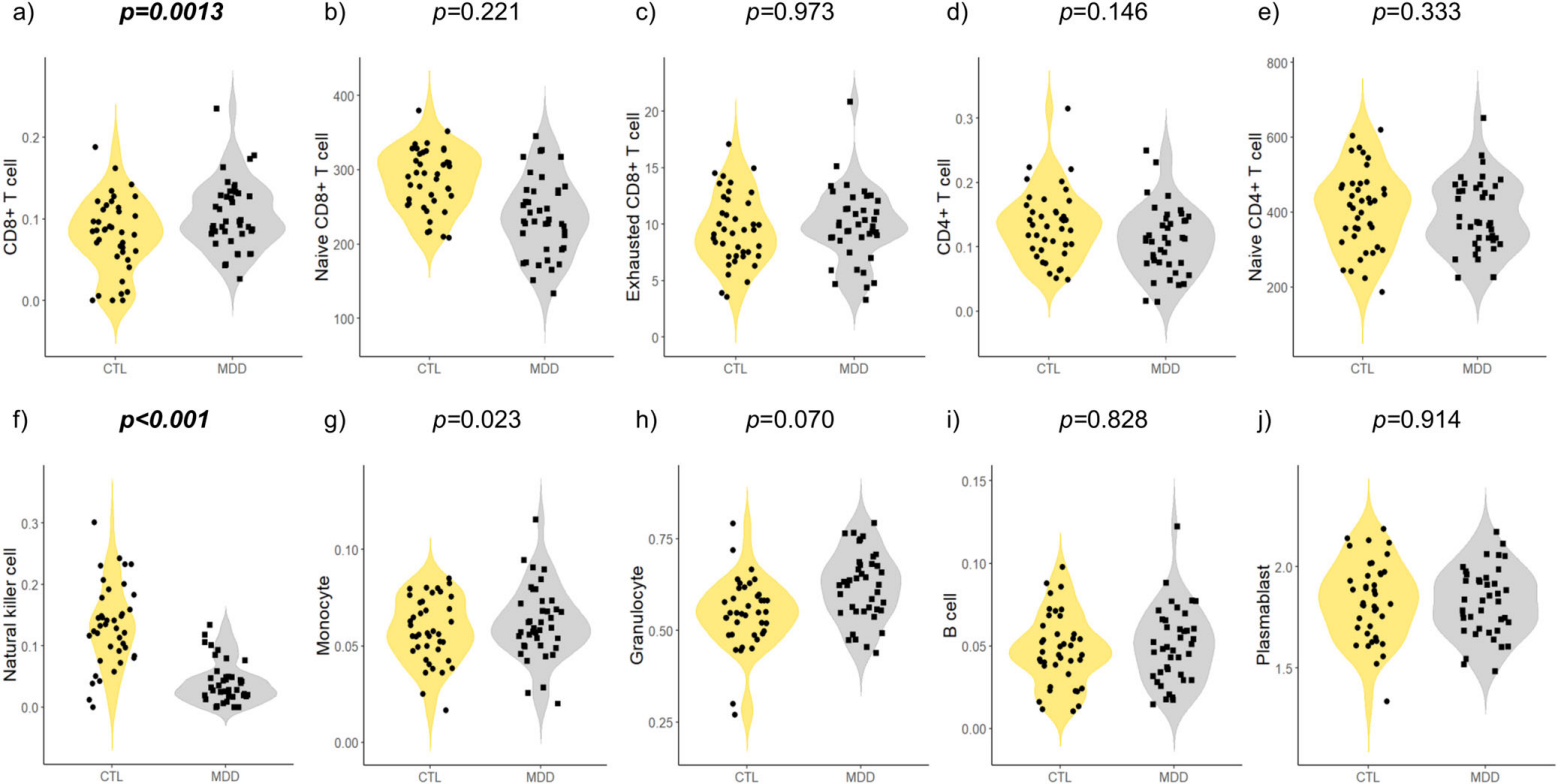
Comparison of DNAm-based white blood cell composition among patients with untreated MDD and controls. **a** cytotoxic CD8+ T cells, (**b**) naive CD8+ T cells, (**c**) exhausted CD8+ T cells, (**d**) helper CD4+ T cells, (**e**) naive CD4+ T cells, (**f**) natural killer cells, (**g**) monocytes, (**h**) granulocytes, (**i**) B cells, and (**j**) plasma blasts. Violin plots with dots indicate the DNAm-based white blood cell composition in patients with untreated MDD and control groups; *p*-values were evaluated using multiple linear regression analysis. We adjusted the significance level for multiple comparisons and the Bonferroni method defined the *p*-value as 0.05/10 = 0.005. If *p*-values are significant at <0.005, they are denoted in bold and italics. CD4, cluster of differentiation 4; CD8, cluster of differentiation 8; CTL, control; DNAm, DNA methylation; MDD, major depressive disorder.

The analysis of DNAm-based white blood cell composition revealed significant differences in CD8+ T cells (*p* = 0.031), naive CD8+ T cells (*p* < 0.001), natural killer (NK) cells (*p* < 0.001), and granulocytes (*p* < 0.001) (Table 1). After adjusting for confounding factors such as age and sex, patients with untreated MDD were associated with an increase in CD8+ T cells (*p* = 0.0013, *R*^2^ = 0.122) and a decrease in NK cells (*p* < 0.001, *R*^2^ = 0.417) (Table 2 and Fig. 3). These results withstood the Bonferroni correction for multiple comparisons of 10 white blood cell compositions (corrected significance was defined as *p*-value < 0.05/10 = 0.005).

### Subgroup analysis of the participants younger than 40 years old

We conducted a subgroup analysis of the participants younger than 40 years old using the same methods and procedures as at the time of analysis for all ages (total analysis), whose age was not significantly different between the control and MDD groups (Age [years old], median [IQR]; CTL, 24.0 [23.0, 26.0]; MDD, 28.0 [24.0, 33.0]; *p* = 0.074) (Table 3 and Supplementary Table 4). Regarding the differences between groups in Table 1 and Table 3, there are no longer significant differences in the three GrimAge components (DNAmGDF-15, *p* = 0.0537; DNAmLeptin, *p* = 0.0776; DNAmPACKYRS, *p* = 0.0942) and one DNAm-based white blood cell composition (Naive CD8+ T cell, *p* = 0.215) (Table 3). The following results were significant in the total analysis and in the analysis of individuals under 40 after adjusting for confounding factors such as age and sex: AgeAccelHannum (*p* < 0.001, *R*^2^ = 0.191), AgeAccelGrim (*p* = 0.0016, *R*^2^ = 0.246), DNAmADM (*p* = 0.00287, *R*^2^ = 0.702), DNAmPAI-1 (*p* = 0.00304, *R*^2^ = 0.186), and DNAmTIMP-1 (*p* = 0.00089, *R*^2^ = 0.867), an increase in CD8+ T cells (*p* = 0.0012, *R*^2^ = 0.161) and a decrease in NK cells (*p* < 0.001, *R*^2^ = 0.361) (Supplementary Table 4).

**Table 3:** Demographics, DNAm-based Age acceleration and telomere length acceleration, as well as GrimAge components, and DNAm-based white blood cell counts between patients with untreated MDD and controls younger than 40 years old.

	CTL (*n* = 39)	MDD (*n* = 17)	*P*-value
*Demographic characteristics*			
Sex (male/female)	20/19	6/11	0.270^a^
Age (years old), median (IQR)	24.0 (23.0, 26.0)	28.0 (24.0, 33.0)	0.074^b^
*DNAmAge and DNAmTL acceleration*^c^			
AgeAccelHorvath, median (IQR)	−0.65 (−2.55, 1.51)	−1.79 (−3.14, 0.62)	0.437^b^
AgeAccelHannum, median (IQR)	−1.77 (−5.22, 1.33)	2.81 (0.62, 4.62)	***<0.001***^***b***^
AgeAccelSkinBlood, median (IQR)	−0.92 (−3.63, 0.62)	1.24 (−0.21, 1.84)	***0.0053***^***b***^
AgeAccelPheno, median (IQR)	−0.86 (−5.11, 2.41)	0.96 (0.35, 4.33)	***0.0346***^***b***^
AgeAccelGrim, median (IQR)	−0.97 (−2.42, 0.50)	1.26 (0.10, 2.54)	***0.0056***^***b***^
DNAmTLadjAge, median (IQR)	0.04 (−0.15, 0.17)	−0.03 (−0.15, 0.12)	0.202^b^
*GrimAge components*^d^			
DNAmADM [pg/ml] (IQR)	276.065 (262.423, 290.016)	299.869 (287.251, 306.975)	***<0.001***^***b***^
DNAmB2M [pg/ml] (IQR)	1114951 (1071275, 1167391)	1218955 (1126147, 1254064)	***0.0037***^***b***^
DNAmCystatinC [pg/ml] (IQR)	503318 (486833, 514898)	521895 (501579, 549532)	***0.0151***^b^
DNAmGDF-15 [pg/ml] (IQR)	272.530 (226.567, 329.455)	365.640 (251.907, 419.807)	0.0537^b^
DNAmLeptin [pg/ml] (IQR)	4950.72 (−1525.32, 6312.50)	5688.32 (−416.632, 7165.86)	0.0776^b^
DNAmPAI-1 [pg/ml] (IQR)	12743.0 (12051.9, 14162.8)	14511.1 (13645.2, 16057.6)	***0.0012***^b^
DNAmTIMP-1 [pg/ml] (IQR)	28629.4 (28296.1, 29020.4)	29691.4 (28608.7, 30386.9)	***0.0084***^***b***^
DNAmPACKYRS (IQR)	5.19338 (2.54860, 6.85158)	8.09970 (4.03462, 10.90834)	0.0942^b^
*DNAm-based white blood cell composition*^e^			
CD8+ T cell (IQR)	0.085 (0.057, 0.110)	0.127 (0.090, 0.145)	***0.0026***^***b***^
Naive CD8+ T cell (IQR)	290.4 (256.1, 322.3)	271.1 (242.7, 296.7)	0.215^b^
Exhausted CD8+ T cell (IQR)	9.47 (7.347, 12.009)	9.159 (5.909, 10.368)	0.447^b^
CD4+ T cell (IQR)	0.132 (0.096, 0.160)	0.106(0.079, 0.141)	0.208^b^
Naive CD4+ T cell (IQR)	418.9 (327.4, 468.0)	456.6 (354.0, 476.2)	0.264^b^
Natural killer cell (IQR)	0.132 (0.094, 0.168)	0.026 (0.017, 0.047)	***<0.001***^***b***^
Monocyte (IQR)	0.057 (0.048, 0.073)	0.060 (0.054, 0.080)	0.228^b^
Granulocyte (IQR)	0.548 (0.490, 0.590)	0.623 (0.564, 0.645)	***0.0261***^***b***^
B cell (IQR)	0.046 (0.038, 0.060)	0.045 (0.032, 0.061)	1.000^b^
Plasmablast (IQR)	1.814 (1.684, 1.964)	1.857 (1.768, 1.946)	0.888^b^

^a^We assessed *p* -value using the χ^2^-test.

^b^We assessed *p* -value using the Mann–Whitney U test.

^c^We defined epigenetic age acceleration (AgeAccelHorvath, AgeAccelHannum, AgeAccelSkinBlood, AgeAccelPheno, and AgeAccelGrim) as the residual from regressing each DNAm age on the chronological age. We defined DNAmTLadjAge as the residual from regressing DNAmTL on chronological age.

^d^GrimAge components indicated DNAm-based age-predictive factors.

^e^These white blood cell counts are predicted by DNAm.

If *p*-values are significant at <0.05, they are denoted in bold and in italics.

*ADM* adrenomedullin, *B2M* beta-2-microglobulin, *CD4* cluster of differentiation 4, *CD8* cluster of differentiation 8, *CTL* control, *DNAm* DNA methylation, *DNAmPACKYRS* DNA methylation-based smoking pack-years, *DNAmTL* DNA methylation-based telomere length, *DNAmTLadjAge* age-adjusted estimate of DNA methylation-based telomere length, *GDF-15* growth differentiation factor 15, *IQR* interquartile range, *MDD* major depressive disorder, *PAI-1* plasminogen activator inhibitor-1, *TIMP-1* tissue inhibitor ofmetalloproteinases-1.

## Discussion

In this study, we provide evidence of epigenetic aging using five epigenetic age clocks, DNAmTL, and GrimAge components, and DNAm-based white blood cell composition in patients with untreated MDD. We found that epigenetic aging acceleration was significantly related to patients with untreated MDD in HannumAge and GrimAge after adjusting for confounding factors such as age and sex. These results are consistent with those of a previous study using GrimAge^23^. Similarly, after adjusting for confounding factors, patients with untreated MDD showed an increase in three GrimAge components (DNAmADM, DNAmPAI-1, and DNAmTIMP-1) as well as an increase in CD8+ T cells and a decrease in NK cells.

Our findings revealed that patients with MDD who had never received depression medication presented with accelerated epigenetic aging using HannumAge and GrimAge epigenetic clocks. In one study, patients who had undergone treatment for MDD had accelerated epigenetic aging as calculated using methods similar to HorvathAge^32^ but showed no significant difference in the five epigenetic clocks^21^. A postmortem study found no significant differences in epigenetic aging acceleration in patients with MDD versus controls using HorvathAge^25^. Another study found accelerated epigenetic aging in MDD compared to controls using peripheral blood based on HorvathAge and GrimAge^20,23^. However, accelerated epigenetic aging differs depending on the patient background as well as on the epigenetic clocks and sample used, and further studies with detailed patient background data are required using the five epigenetic clocks.

In addition, we observed a tendency for shorter telomere length based on DNAmTL when we conducted the analysis through all-age. Telomeres are unique structures located on the terminals of chromosomes, required to protect chromatin from DNA damage^35^. Telomeres shorten through cell replication, and telomere shortening is considered to signify a “molecular clock” based on underlying cell aging, which can render the body more vulnerable to diseases related to aging^35–37^. Patients with chronic MDD presented with significantly shorter leukocyte telomere lengths (LTL) than healthy controls^38^. The number of depressive episodes has been reported to be associated with a shorter LTL^39^. Furthermore, patients with untreated MDD have significantly shorter telomeres compared to those of controls^40^. A previous study indicated that antidepressant medication may protect against telomere shortening^8^. Lu et al. demonstrated that DNAmTL exhibited a stronger correlation with chronological age than did LTL, and it outperformed LTL in predicting both morbidity and mortality^14^. We suggest that depression is associated with accelerated biological aging, leading to premature mortality from various diseases. Chronic stress leads to dysregulation of the hypothalamic-pituitary adrenal (HPA) axis, resulting in the development of depression and various organ disorders^41^. HPA axis dysregulation is associated with cardiovascular mortality and many cardiovascular disease risk factors such as obesity, hypercholesterolemia, hypertriglyceridemia, elevated blood pressure, and diabetes^42^. Although antidepressants may inhibit HPA activity, reduce levels of cortisol, and prevent the development of various diseases, further studies are needed to elucidate the mechanism by which antidepressants reduce epigenetic aging in patients with MDD^43^.

We found that DNAmADM, DNAmPAI-1, and DNAmTIMP-1 levels were increased and DNAmCystatinC is likely to increase in patients with untreated MDD, suggesting that these epigenetic markers may be useful in understanding the pathophysiology of MDD and as potential biomarkers. Previous studies have also reported an association between increased levels of DNAmCystatinC and MDD^21,23^, as well as higher serum cystatin C levels in individuals with depression^44–46^. Cystatin C inhibits endogenous cysteine protease activity, is an established marker of kidney function, and involved in immunomodulation and inflammation^47,48^. A large number of studies have also reported that inflammation is one of the pathogenic factors in MDD^49–51^. In addition, cystatin C is involved in the signal transduction pathway of interferon-gamma (IFN-γ), which is predominantly secreted by NK and T cells^48^.

We observed a marked decrease in DNAm-based NK cells in patients with untreated MDD. Chronic stress reduces NK cells in the bone marrow and blood of mice and has been reported to also decrease NK cell counts in humans^52,53^. Patients with MDD had lower levels of CD56^+^CD16^−^ NK cells, which are the major subtype producing IFN-γ, as well as lower IFN-γ levels compared to healthy controls^54–56^. This suggests a possible association between NK cells, cystatin C, and the development of depression due to inflammation. In contrast, previous studies have shown that increased DNAm-based NK cells are related to suicide completion, which is one of the most severe phenotypes of depression^29^. Additionally, acute psychological stress has been found to increase NK cells^57^. These findings suggest that acute psychological stress leading to suicide increases NK cells, whereas chronic stress decreases NK cells. Further studies of NK cells may help us understand the severity of depression in patients with MDD.

A previous study showed that the severity of MDD is associated with a lower cluster of differentiation 4 (CD4)+/ cluster of differentiation 8 (CD8) ratio, which indicates that its severity is linked to a decrease in CD4+ cells and an increase in CD8+ cells, which is consistent with our results^58^. While the immune-inflammatory hypothesis of depression is supported by accumulating evidence, it remains inconclusive and requires further research^49–51^.

As for other GrimAge components that we found to be associated with MDD, ADM is thought to play a regulatory and protective role in the hypothalamic-pituitary-adrenal (HPA) axis activated by stressors^59^. Patients with MDD had significantly higher serum ADM concentrations than did healthy controls^60^. Moreover, patients with MDD had significantly higher serum levels of PAI-1 and TIMP-1 than did controls^61–63^. Although our findings are consistent with those of previous studies, further research is required to confirm reproducibility of our results with larger sample sizes using actual plasma and serum to elucidate the pathophysiology of MDD and develop biomarkers.

Our study had several limitations. First, our sample size was small and only whole-blood samples were used. Second, the GrimAge components estimate plasma levels based on DNAm profiles and require verification using actual plasma. This is equally true for DNAm-based white blood cell composition. Third, we did not analyze other confounding factors related to the DNAm profile, such as the participant’s medical history, smoking history, and adverse childhood experiences, which impacted our results. Fourth, the control group was much younger than the MDD group. We did perform subgroup analysis of the participants younger than 40 years old, but this analysis halved MDD group and was targeted to people of a specific age. Although we adjusted our results for age as much as we could, a bias could still have remained and affected our conclusions. We also need to be cautious about interpreting the results of DNAmTL and DNAmCystatinC, where significant differences disappeared in the subgroup analysis limited to those under 40 years of age. Further study, with no age limit, matched for age and with an increased sample size, is required. Finally, we could not compare treated patients with MDD and untreated patients in the study to directly evaluate the effects of antidepressants based on the links between MDD and epigenetic age.

In conclusion, we investigated epigenetic aging using five epigenetic clocks, DNAmTL, GrimAge components, and DNAm-based white blood cell composition in patients with untreated MDD. We found a significant tendency for association between patients with untreated MDD and accelerated epigenetic aging, as determined by HannumAge and GrimAge. Additionally, our findings suggest that patients with untreated MDD are associated with alterations in several DNAm-based plasma proteins and two DNAm-based white blood cell composition, including decreased NK cells. It is possible that these findings provide insight into the pathophysiology and biomarkers of MDD, as well as unknown mechanisms of action of antidepressants.

## Methods

### Sample datasets (GSE201287)

In the present study, publicly available DNAm data was utilized from the Gene Expression Omnibus database. The GSE201287 dataset was contributed by Han and Liu et al. and comprised patients with untreated MDD (*n* = 40) and controls (*n* = 40), who gave written informed consent to attend the study approved by the Ethics Committee of the Chinese Academy of Medical Sciences and the Peking Union Medical College. All the subjects were of Chinese Han origin and recruited at the Department of Psychiatry, First Hospital of Shanxi Medical University, Taiyuan, China. All patients with MDD were in acute depressive episodes, with no history of psychiatric or psychological visits, and had not previously received treatment with medication. All patients were evaluated by at least two psychiatrists based on the Diagnostic and Statistical Manual of Mental Disorders Fourth Edition (DSM- IV). They used the Illumina Infinium 450k Human DNA methylation Beadchip data to obtain genome-wide DNA methylation profiles in peripheral blood samples (GEO Accession viewer (nih.gov))^64^.

### Estimation via five epigenetic clocks, DNAmTL, and GrimAge components

Five epigenetic clocks (HorvathAge, HannumAge, SkinBloodAge, PhenoAge, and GrimAge) and DNAmTL were utilized via an online DNAm age calculator in the present study (https://horvath.genetics.ucla.edu/html/dnamage/)^10^. First-generation epigenetic clocks, HorvathAge and HannumAge, were developed by Horvath and Hannum et al.^9,10^. Horvath et al. developed SkinBloodAge as a revised clock using skin and blood cells to compensate for lower accuracy of first-generation epigenetic clocks in terms of fibroblasts^11^. Subsequently, PhenoAge, GrimAge, and DNAmTL have also been created, which have superior features in terms of predicting mortality and various health risks compared with first-generation epigenetic clocks^12–14^. GrimAge components comprise chronological age, sex, seven DNAm-based age-predictive plasma proteins (adrenomedullin [ADM], beta-2-microglobulin [B2M], cystatin C, growth differentiation factor-15 [GDF-15], leptin, plasminogen activation inhibitor-1 [PAI-1], and tissue inhibitor of metalloproteinases-1 [TIMP-1]), and DNAmethylation-based smoking pack-years (DNAmPACKYRS) in order to function as an epigenetic clock^13^. Epigenetic age acceleration (AgeAccelHorvath, AgeAccelHannum, AgeAccelSkinBlood, AgeAccelPheno, and AgeAccelGrim) means the residual from regressing each DNAm age on chronological age. Positive and negative values signify whether the epigenetic age is higher or lower comparison with the anticipated age (determined by chronological age). The age-adjusted estimate of DNAm-based TL (DNAmTLadjAge) is the residual calculated by regressing DNAmTL on the chronological age. Positive and negative values signify whether DNAmTLadjAge was longer or shorter than the anticipated DNAmTL (determined by chronological age), respectively. GrimAge components are described in this paper as follows: DNAmADM, DNAmB2M, DNAmCystatinC, DNAmGDF-15, DNAmleptin, DNAmPAI-1, DNAmTIMP-1, and DNAmPACKYRS^13^.

### White blood cell composition predicted based on DNAm

Houseman et al. and Horvath developed the DNAm-based white blood cell composition^10,15^. We performed analysis of DNAm-based white blood cell composition using an online DNAm age calculator (https://horvath.genetics.ucla.edu/html/dnamage/)^10^. The DNAm-based white blood cell composition comprises a cytotoxic CD8+ T cells, naive CD8+ T cells, exhausted CD8+ T cells, helper CD4+ T cells, naive CD4+ T cells, NK cells, monocytes, granulocytes, B cells, and plasma blasts, which are estimated based on DNAm using Houseman’s and Horvath’s method^10,15^.

### Statistical analysis

The data were analyzed using R version 4.2.2 (R Development Core Team, Vienna, Austria) and EZR software (version 1.61; Jichi Medical University, Saitama, Japan)^65^. We analyzed categorical variables and between-group differences in continuous variables using the χ^2^-test and Mann–Whitney *U* test, respectively. Regarding confounding factors, such as age and sex, we conducted multiple linear regression analyses. To examine the relationship between continuous variables, we performed Spearman’s rank correlation coefficient. Dummy variables were used where needed. Statistical significance was defined as a two-tailed *p*-value < 0.05.

### Reporting summary

Further information on research design is available in the Nature Research Reporting Summary linked to this article.

### Supplementary information

Suppleymentary Material

Reporting Summary

## Data Availability

We utilized the GSE201287 dataset that is publicly available from the Gene Expression Omnibus database (GEO Accession viewer (nih.gov)), which has been used in previous studies^64^.
