# Supplementary material for: Accelerated epigenetic aging and decreased natural killer cells based on DNA methylation in patients with untreated major depressive disorder
Source: NPJ Aging. 2023 Sep 6;9(1):19. doi: 10.1038/s41514-023-00117-1 (PMC10482893; doi:10.1038/s41514-023-00117-1)
Supplement: Supplementary file 1 — Suppleymentary Material [file 41514_2023_117_MOESM1_ESM.pdf]

Supplementary material

Accelerated epigenetic aging and decreased natural killer cells based on DNA methylation in untreated patients with major depressive disorder

Ryota Shindo [1 †], Takaki Tanifuji [1 †], Satoshi Okazaki [1] *, Ikuo Otsuka [1], Toshiyuki Shirai [1], Kentaro Mouri [1], Tadasu Horai [1], and Akitoyo Hishimoto [1]

[a] Department of Psychiatry, Kobe University Graduate School of Medicine, Kobe, Japan

[†] These authors equally contributed to the work.

* **Corresponding to**: Satoshi Okazaki, B.S., M.D., Ph.D. (okazakis@med.kobe-u.ac.jp)

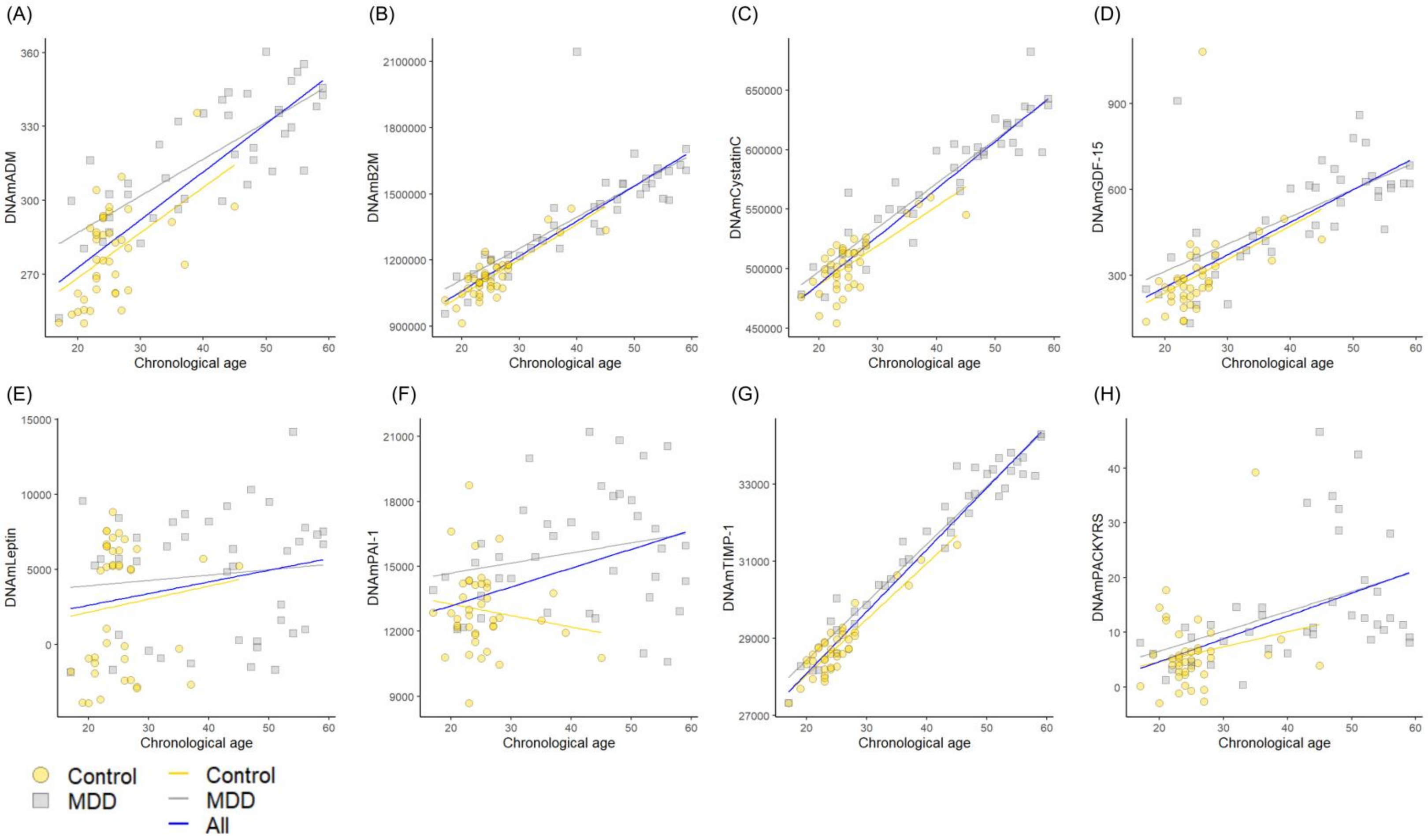

Supplementary Figure 1. Correlation between GrimAge components and chorological age among patients with untreated MDD and controls.

(A) ADM, (B) B2M, (C) Cystatin C, (D) GDF-15, (E) Leptin, (F) PAI-1, (G) TIMP-1, and (H) PACKYRS. The scatter plots indicate GrimAge components on the y-axis vs. the chronological age on the x-axis.

ADM, adrenomedullin; BMI, body mass index; B2M, beta-2-microglobulin; CTL, control; DNAm, DNA methylation; GDF-15, growth differentiation factor 15; MDD, major depressive disorder; PACKYRS, smoking pack-years; PAI-1, plasminogen activator inhibitor-1; TIMP-1, tissue inhibitor ofmetalloproteinases-1.

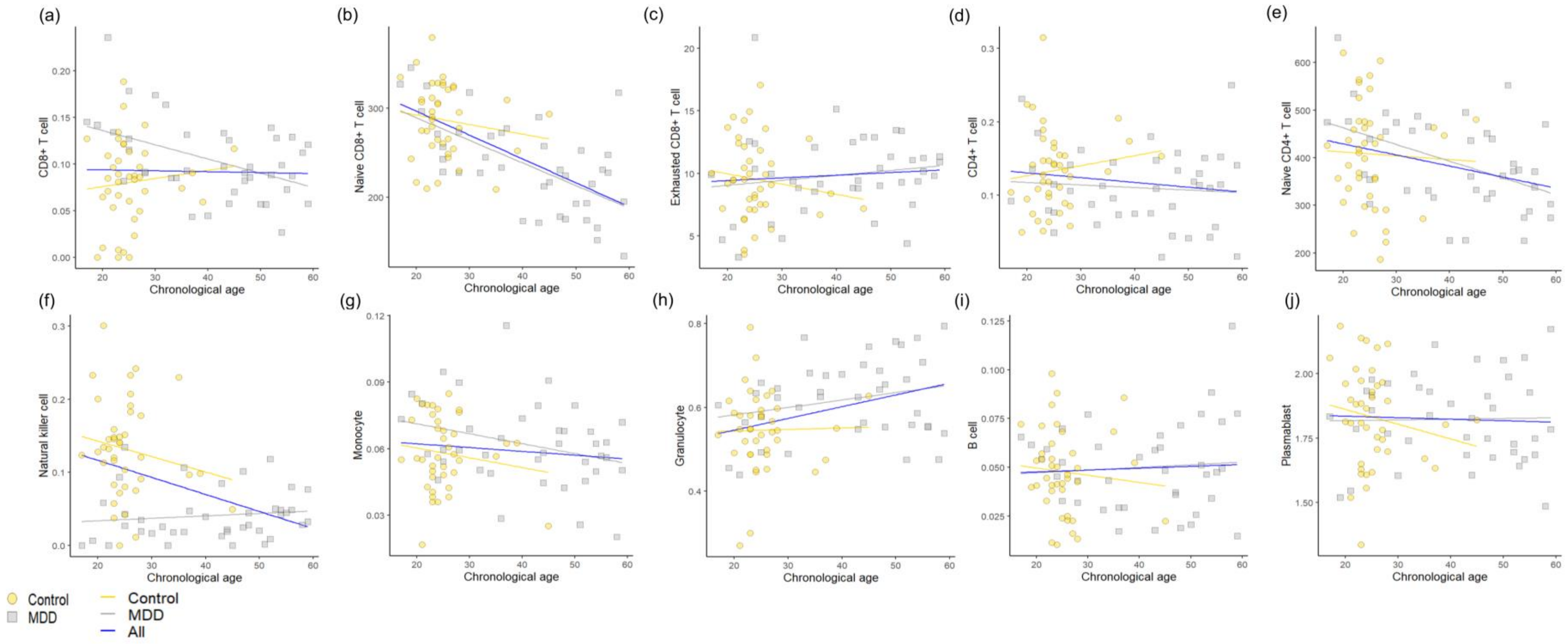

Supplementary Figure 2. DNAm-based white blood cell composition correlated with the chronological age among patients with untreated MDD and controls.

(a) cytotoxic CD8+ T cells, (b) naive CD8+ T cells, (c) exhausted CD8+ T cells, (d) helper CD4+ T cells, (e) naive CD4+ T cells, (f) natural killer cells, (g) monocytes, (h) granulocytes, (i) B cells, and (j) plasma blasts.

White blood cell counts were estimated using DNAm. Scatter plots indicate DNAm-based white blood cell composition versus chronological age (*p*-values were evaluated using Spearman's rank correlation coefficient).

CD4, cluster of differentiation 4; CD8, cluster of differentiation 8; CTL, control; DNAm, DNA methylation; MDD, Major depressive disorder.

Supplementary Table 1. Correlation between DNAm-based Age and chorological age in patients with untreated MDD and controls

	Phenotype	*rho*	*p*-value
**HorvathAge**	CTL	0.709	***<0.001***
	MDD	0.873	***<0.001***
	ALL	0.896	***<0.001***
**HannumAge**	CTL	0.602	***<0.001***
	MDD	0.877	***<0.001***
	ALL	0.876	***<0.001***
**SkinBloodAge**	CTL	0.818	***<0.001***
	MDD	0.893	***<0.001***
	ALL	0.924	***<0.001***
**PhenoAge**	CTL	0.519	***0.0006***
	MDD	0.879	***<0.001***
	ALL	0.857	***<0.001***
**GrimAge**	CTL	0.668	***<0.001***
	MDD	0.883	***<0.001***
	ALL	0.908	***<0.001***
**DNAmTL**	CTL	− 0.0332	0.839
	MDD	− 0.816	***<0.001***
	ALL	− 0.721	***<0.001***

We assessed *p* -value using the Spearman's rank correlation coefficient.

If *p*-values are significant at <0.05, they are denoted in bold and in italics.

CTL, control; DNAmTL, DNA methylation-based telomere length MDD, Major depressive disorder

Supplementary Table 2. Correlation between GrimAge components and chorological age in patients with untreated MDD and controls

	Phenotype	*rho*	*p*-value
**DNAmADM**	CTL	0.507	***0.00085***
	MDD	0.763	***<0.001***
	ALL	0.791	***<0.001***
**DNAmB2M**	CTL	0.677	***<0.001***
	MDD	0.886	***<0.001***
	ALL	0.899	***<0.001***
**DNAmCystatinC**	CTL	0.633	***<0.001***
	MDD	0.917	***<0.001***
	ALL	0.894	***<0.001***
**DNAmGDF-15**	CTL	0.628	***<0.001***
	MDD	0.688	***<0.001***
	ALL	0.774	***<0.001***
**DNAmLeptin**	CTL	0.112	0.491
	MDD	0.132	0.417
	ALL	0.244	***0.0293***
**DNAmPAI-1**	CTL	− 0.119	0.465
	MDD	0.198	0.220
	ALL	0.375	***0.0006***
**DNAmTIMP-1**	CTL	0.752	***<0.001***
	MDD	0.949	***<0.001***
	ALL	0.940	***<0.001***
**DNAmPACKYRS**	CTL	0.0453	0.781
	MDD	0.509	***0.0008***
	ALL	0.537	***<0.001***

We assessed *p* -value using the Spearman's rank correlation coefficient.

If *p*-values are significant at <0.05, they are denoted in bold and in italics.

ADM, adrenomedullin; B2M, beta-2-microglobulin; CTL, control; DNAm, DNA methylation; DNAmPACKYRS, DNA methylation-based smoking pack-years; GDF-15, growth differentiation factor 15; MDD, Major depression disorder; PAI-1, plasminogen activator inhibitor-1; TIMP-1, tissue inhibitor ofmetalloproteinases-1

Supplementary Table 3. DNAm-based white blood cell composition correlated with the chronological age in patients with untreated MDD and controls

	Phenotype	*rho*	*p*-value
**CD8+ T cell**	CTL	0.095	0.558
	MDD	− 0.394	***0.012***
	ALL	− 0.027	0.810
**Naive CD8+ T cell**	CTL	− 0.132	0.416
	MDD	− 0.588	***<0.001***
	ALL	− 0.598	***<0.001***
**Exhausted CD8+ T cell**	CTL	− 0.170	0.293
	MDD	0.216	0.181
	ALL	0.089	0.431
**CD4+ T cell**	CTL	0.089	0.586
	MDD	− 0.0464	0.776
	ALL	− 0.131	0.245
**Naive CD4+ T cell**	CTL	− 0.107	0.513
	MDD	− 0.432	***0.005***
	ALL	− 0.289	***0.009***
**Natural killer cell**	CTL	− 0.142	0.383
	MDD	0.259	0.107
	ALL	− 0.386	***0.0004***
**Monocyte**	CTL	− 0.016	0.921
	MDD	− 0.291	0.068
	ALL	− 0.092	0.417
**Granulocyte**	CTL	− 0.023	0.890
	MDD	0.162	0.318
	ALL	0.332	0.003
**B cell**	CTL	− 0.203	0.208
	MDD	0.041	0.801
	ALL	− 0.076	0.503
**Plasmablast**	CTL	− 0.141	0.385
	MDD	− 0.0358	0.827
	ALL	− 0.040	0.724

We assessed *p* -value using the Spearman's rank correlation coefficient.

If *p*-values are significant at <0.05, they are denoted in bold and in italics.

CD4, cluster of differentiation 4; CD8, cluster of differentiation 8; CTL, control; DNAm, DNA methylation; MDD, Major depressive disorder

Supplementary Table 4. Multiple regression analyses of DNAm-based Ages and TL acceleration, as well as GrimAge components, and DNAm-based white blood cell counts between patients with untreated MDD and controls younger than 40 years old

	Phenotype		Age		Sex		Adjusted R^2
	Estimate	*P*-value	Estimate	*P*-value	Estimate	*P*-value	
**DNAmAge and DNAmTL acceleration** [a]							
AgeAccelHorvath	− 0.94919606	0.500	0.07602254	0.546	− 1.97439809	0.119	0.007486
AgeAccelHannum	5.38458490	***<0.001***	− 0.02734922	*0.835*	− 2.41054733	0.069	0.1914
AgeAccelSkinBlood	0.9996374	0.461	0.1516941	0.214	− 1.0495608	0.386	0.004659
AgeAccelPheno	2.5288128	0.182	0.1213498	0.471	− 2.0435853	0.226	0.01807
AgeAccelGrim	2.295827438	***0.0016***	0.003072431	0.960	− 2.129536659	***0.00106***	0.2458
DNAmTLAdjAge	− 0.077913526	0.131	0.001547362	0.734	0.101123630	***0.030***	0.06086
**GrimAge components** [b]							
DNAmADM	10.702830	***0.00287***	1.908057	***<0.001***	22.206395	***<0.001***	0.7027
DNAmB2M	25007.25	0.159	17763.46	***<0.001***	15923.37	0.312	0.7335
DNAmCystatinC	11414.879	0.0344	3778.947	***<0.001***	− 2245.985	0.633	0.5968
DNAmGDF-15	39.393067	0.407	9.519754	0.028	15.606152	0.712	0.0792
DNAmLeptin	420.6267	0.442	28.8737	0.554	7574.7376	***<0.001***	0.8225
DNAmPAI-1	1798.56551	***0.00304***	71.92594	0.170	− 738.19550	0.158	0.1862
DNAmTIMP-1	381.0633	***0.00089***	165.3476	***<0.001***	− 178.9794	0.0687	0.8668
DNAmPACKYRS	0.9295317	0.611	0.4119869	0.0143	− 3.5397149	0.0334	0.1353
**DNAm-based white blood cell composition** [c]							
CD8+ T cell	0.04777	***0.0012***	− 0.00156	0.219	0.01117	0.375	0.1611
Naive CD8+ T cell	− 7.6774	0.525	− 2.5083	0.0234	7.4340	0.490	0.07248
Exhausted CD8+ T cell	− 0.36848	0.734	− 0.01344	0.890	− 0.25152	0.795	− 0.05201
CD4+ T cell	− 0.01841	0.268	− 0.00027	0.856	0.00234	0.873	− 0.02812
Naive CD4+ T cell	33.801	0.259	− 4.9206	0.0687	67.541	0.0134	0.1238
Natural killer cell	− 0.09080	***<0.001***	− 0.00017	0.915	− 0.02590	0.109	0.3614
Monocyte	0.00917	0.094	− 0.00008	0.862	− 0.00680	0.162	0.02481
Granulocyte	0.04360	0.131	0.00262	0.307	0.02301	0.367	0.05322
B cell	0.00152	0.806	− 0.00068	0.228	− 0.00453	0.414	− 0.01306
Plasmablast	0.00701	0.903	0.00135	0.794	− 0.01865	0.718	− 0.05333

Dummy variables: phenotype, CTL = 0, and MDD = 1; sex, male = 0 and female= 1.

If *p*-values are significant at <0.05, they are denoted in bold and in italics.
R^2 is the coefficient of determination.
[a] We defined epigenetic age acceleration (AgeAccelHorvath, AgeAccelHannum, AgeAccelSkinBlood, AgeAccelPheno, and AgeAccelGrim) as the residual from regressing each DNAm age on the chronological age. We defined DNAmTLadjAge as the residual from regressing DNAmTL on chronological age.
[b] GrimAge components indicated DNAm-based age-predictive factors. For the GrimAge components, we corrected the significance level for multiple comparisons and the Bonferroni method defined the *p*-value as 0.05/8 = 0.00625 (eight GrimAge components).
If *p*-values are significant at <0.00625, they are denoted in bold and in italics.
[c] These white blood cell counts were predicted using DNAm. For DNAm-based white blood cells, we adjusted the significance level for multiple comparisons and the Bonferroni method defined the *p*-value as 0.05/10 = 0.005 (ten white blood cell compositions).
If *p*-values are significant at <0.005, they are denoted in bold and in italics.
ADM, adrenomedullin; B2M, beta-2-microglobulin; CD4, cluster of differentiation 4; CD8, cluster of differentiation 8; CTL, control; DNAm, DNA methylation; DNAmPACKYRS, DNA methylation-based smoking pack-years; DNAmTL, DNA methylation-based telomere length; DNAmTLadjAge, age-adjusted estimate of DNA methylation-based telomere length; GDF-15, growth differentiation factor 15; MDD, Major depressive disorder; PAI-1, plasminogen activator inhibitor-1; TIMP-1, tissue inhibitor ofmetalloproteinases-1
